# Design Models for Single Patient Rooms Tested for Patient Preferences

**DOI:** 10.1177/1937586720937995

**Published:** 2020-07-14

**Authors:** Clarine J. van Oel, Meloek Mlihi, Arno Freeke

**Affiliations:** 1 Department of Architecture and the Built Environment, 2860 Delft University of Technology, The Netherlands

**Keywords:** evidence-based design, hospital design, patient preferences, discrete choice experiments, single patient rooms

## Abstract

Using 3D design models, this study aims to better involve patients in the design of hospitals by investigating what physical environmental characteristics in hospital patient rooms are valued by patients. There is a plea for shared-decision-making and collaborative design processes with representatives from healthcare and the construction sector based on evidence and end users’ perspectives. Existing research is hampered by poor conceptualization of environmental design factors, as these are differently operationalized between medical and technological sciences. Architects communicate through visuals, whereas medical professionals and researchers tend to communicate in words. By using 3D-modeling to research the relationship between health and well-being on the one hand, and the affordances the built environment offers, this knowledge gap can be better addressed. Two hundred four respondents, 60% patients and 40% medical professionals, engaged in discrete choice experiments visualizing a single patient room. A main finding is that patients and medical professionals consistently choose for hospital rooms with the highest amount of daylight access. What this study adds is that the orientation of the windows matters as well. Horizontal windows, allowing for a panoramic view, were twice as much chosen than were vertical windows. Another important finding concerns patients’ preferences for an open door, suggesting patients prefer to stay “connected” to the outside world. This study is important as it shows, empirically, that patients may make different choices if in research the rooms are better conceptualized and thus visualized and if multiple design features are assessed as configuration rather than using a sequential, “one-design-characteristic-after-another” approach.

In 1984, Ulrich published his landmark study in a short report in *Science*, showing earlier discharge from hospital upon surgery among patients with a view to nature through their room’s window as compared to patients who had a view on a blind wall (Ulrich, 1984). With this study, Ulrich founded the movement toward evidence-based design (EBD), which is the theoretical concept of what often is referred to as healing environments (Huisman et al., 2012). The importance of EBD is acknowledged in the planning and (re)design of healthcare facilities (HCF) such as Erasmus MC and by institutes like the Center for Health Design. This landmark study has also received accelerating academic attention including research from architecture and building technology into the impact of physical environmental factors on patients and/or staff (Huisman et al., 2012; Mourshed & Zhao, 2012; Salonen et al., 2012), environmental psychological research particularly targeting the restorative (stress-reducing) effects of supportive design of hospital rooms (Andrade & Devlin, 2015; Ulrich et al., 1991), and the use of gardens as a therapeutic approach (Cimprich & Ronis, 2003; Velarde et al., 2007).

What Ulrich back in 1984 showed was that living environments can provide environmental press (Lawton & Nahemow, 1973) to patients, and this might be particularly true to those with declining cognitive capacities such as elderly. Environmental press arises if the demands of the environment exceed the demands and abilities of a person. The concept of affordances was introduced by Gibson and refers to the cues that a building can offer a person in terms of perception and behavior. Affordances can be considered as the perceptual properties of the hospital that have functional significance for a person (Heft, 2010). Consequently, affordances play a role in cognitive processes such as spatial memory, which might be implicated in people experiencing stress due to hospitalization (Lengen & Kistemann, 2012). Depending on the patient’s cognitive capacities and age, environments that are conventionally designed for the cognitively able appear to put stress on the cognitive abilities of elderly with diminishing cognitive functioning, including people with dementia (Zeisel et al., 2003). However, in people with high cognitive functioning, allocation of attention to novelty facilitates adaption to a changing environment (Daffner et al., 2006) and a more complex design will be therefore perceived as better affording their well-being.

This balancing between well-being and the amount of environmental press that patients may experience during hospitalization or an outpatient visit is often discussed in architecture, for instance, regarding the acoustic environment (Salonen et al., 2013).

However, despite the many guidelines for hospital design that may support well-being, the evidence-base of guidelines in healthcare design is rather poor (Day et al., 2000; Fleming & Purandare, 2010; Goehner et al., 2018; Herweijer-van Gelder, 2016). One problem with existing research is the poor conceptualization of environmental design factors (van Hoof et al., 2010).

## Ill-Defined Concepts

For several reasons, environmental design factors are poorly conceptualized. First, as van Hoof (2010) put forward, the same concepts are operationalized in different ways between medical and technological sciences. To some extent, this is related to a lack of multidisciplinary expertise in (research) teams, since building technology is a different specialism than architecture. Aarts et al. (2016) reviewed the technological quality of the light intervention’s descriptions in phototherapy studies, as complete descriptions are required for proper replication and comparisons across studies, for instance, in meta-analyses. They showed that the technological details fell short in all assessed studies. Secondly, there is a communication problem. Whear et al. (2014), for instance, reported that interventions involving garden design were generally poor as all studies lacked a detailed description of what the garden design constituted. Environmental designs descriptions are complicated as these physical design characteristics are processed and memorized as configurationally information (Hoegg & Alba, 2008; Lengen & Kistemann, 2012). Therefore, environmental designs are better described as visuals than in words (van Oel & van den Berkhof, 2013), and that is how designers work. Architects communicate through visuals, whereas the medical professionals and researchers tend to communicate in words. During the design process, architects translate concepts like a “homelike environment” into a design. However, architects have no strong tradition in patient-centered design processes and typically use design approaches in which they translate user requirements as expressed in the design brief into design proposals.

***For several reasons, environmental design factors are poorly conceptualized. First, as van Hoof (2010)***

***… put forward, the same concepts are operationalized in different ways between medical and technological sciences***.

## Patient-Centered Design Processes

Patient-centered care considers both the physical and psychosocial aspects of the environment. A challenge in healthcare architecture is to truly integrate the needs of patients, informal carers, and professionals into design decisions (Elf et al., 2015). Designing patient-centered care requires thorough analysis of the required spatial conditions that support the care processes (Elf et al., 2015). This part of the process precedes the actual design phase in which sketches of the hospital becomes increasingly more detailed.

Product development differs between the construction and manufacturing industries, as the (re)design and construction of buildings are time-based, site-specific projects with many partners with knowledge from many different technical and nontechnical fields (Eriksson, 2015). That means that in developing the project, parties have to effectively arrange and structure their working processes. Much effort is put into collaboration and integration of activities between project team members to manage costs and time overruns. Typically, quality management systems are used, emphasizing the operational quality of the building process instead of to what extent the delivered project meets the user’s needs (Thomson et al., 2003). Examples of codesign with patients and informal carers are mostly stemming from industrial design (i.e., manufacturing industry, see, for instance, Sanders & Stappers, 2008, 2014; Visser et al., 2005). An important exception is the work of Elf et al. (2016, 2007), though they only included healthcare professionals. However, their use of system dynamics to develop causal loop diagrams in codesign workshops was evaluated as too difficult (Elf et al., 2016; Elf et al., 2007). van Hoof et al.’s In2Health model (van Hoof et al., 2015; van Hoof & Verkerk, 2013) can be used by building project managers (or architects) to keep oversight of the needs of stakeholders, including patients, and is better described as a model guiding the decision-making process such that the final design maximizes key success factors. It is not so much an example of codesign and shared decision making.

## The Current Study

This project aims to better involve patients’ preferences in the design process of a newly built hospital in the Netherlands. Previous research by de Boer-Lootens (2014) and Grinwis (2016) showed that it may be very difficult for family caregivers and medical professionals to stand in the shoes of patients. When asked to indicate what choice a person would made, these choices were different. Therefore, the aim of the current study is to investigate what physical environmental characteristics in hospital patient rooms contribute to the well-being of patients and to investigate whether a proxy matches patients’ choices. More particularly, the following subquestions were (1) What are the physical environmental characteristics preferences of a patient room in the eyes of patients; and (2) In the eyes of medical professionals, what are the patients’ preferences for the physical environmental characteristics of a patient room?

In the remaining, first as part of the background an analysis of healthcare architecture will be presented, followed by a discussion of the methods. Thereafter, the results of the pilot study and the discrete choice experiments (DCEs) will be given. Finally, an answer to the research questions, presented after the background section on healthcare architecture will be given, followed by a discussion and reflection of the main findings. In the closure, recommendations will be made.

## Literature Review

To identify the main topics in healthcare architecture, a search was conducted in Web of Science using a search strategy to identify research on healthy environments published since 2012. This search strategy reads as follows: (TOPIC: ((((housing OR dwelling OR urban OR neighborhood OR hospital OR (nursing OR care) NEAR home) AND ((healthy OR healing) NEAR environment*)) NOT food)) AND YEAR PUBLISHED: (2012-2018) Refined by: RESEARCH AREAS: (CONSTRUCTION BUILDING TECHNOLOGY OR URBAN STUDIES OR ARCHITECTURE).

The results of this search were combined with this search strategy for all publications in the *Health Environments Research & Design Journal*, without the refinement by research areas. Vosviewer was used for text analyses and visualization of the results and identified three clusters. The first cluster can be identified as related to hospital design. This cluster includes items that describe perceptions of the physical environment and patient rooms and factors that may be important to the design of patient rooms such a privacy, lighting, noise, (patient) safety, and infection. Infection maybe a medical outcome, but mold, for instance, is related to air tight control and is an important aspect in designing hospitals. Nature is part of the second cluster which can be interpreted as items that take on a social science perspective. In the third cluster, the more recent focus is on the city, and the healthy environment, which seemed to develop from former research into sustainable development and energy efficiency. Items like energy consumptions, sustainable development alongside energy and indoor environment describe research into energy efficiency and (indoor) climate. The emphasis is on energy efficiency of buildings and (indoor) climate and there is no strong evidence for research into user preferences for architectural design factors in the design process.

### General Design Requirements in Hospital Design

So far, text analyses of scientific titles and abstracts thus showed that noise, lighting, safety, infection, privacy and view to greenery, as well as indoor environment, and the physical environment could be important to patients’ preferences in the design of patient rooms.

As the aim of this study is to investigate what physical environmental characteristics in hospital patient rooms contribute to the well-being of patients, other design factors might be relevant as well. Therefore, an analysis of a design brief of a hospital was made to identify additional factors relevant to the design of patient rooms. In the analysis of the design brief, the following design implications to the present study were identified. First, an important requirement was that all patient rooms were to be single-patient rooms with “individual” sanitary. Furthermore, patients were to have outside view; all patients, visitors and staff should be able to directly get in contact with a natural environment; the design should be senior friendly; orientation should be according to environmental psychological principles, that is, providing overview and natural guidance. Also, the design should reflect a healing environment, thus emphasizing greenery; needs to adhere to the Human Scale; and finally, the design should provide natural transitions from public, semipublic to enclosed and closed spaces.

The design brief emphasized senior-friendly design as an important additional framework. With increasing age, elderly might have increasingly difficulties in “sensory comprehension of spaces,” as such depends on one’s cognitive abilities (Slaughter et al., 2006; Zeisel et al., 2003). Zeisel et al., (2003) studied residential care for people with dementia environments and suggested that environments conventionally designed for the cognitively able appear to put stress on their cognitive abilities. The challenge thus is to design restorative environments and many studies show that nature is an important element in environments that promote the renewal of adaptive resources (von Lindern et al., 2017). Indeed, the importance of nature in dealing with stress is also emphasized in the design brief in requesting a healing environment as well as that patients need to have a direct outside view and that patients, visitors, and staff should be able to directly get into contact with nature. Nature is a key concept in those requirements, and this is consistent with nature being one of the important and more recently studied concepts in the second cluster.

## Method

The here described study was designed as a mixed-method study to examine the relationship between physical place characteristics and well-being in hospital settings to develop user-preference tested design models for patient rooms.

### Pilot Study

Since the literature review identified no detailed design characteristics, but only directions of inquiry, a long list of design characteristics was developed (see Table 1). This long list was then assessed for the relevance of individual design attributes by experts using an online survey. The outcomes of this survey were discussed with an expert panel in a focus group setting, and the outcomes were used to decide about a short list of design characteristics that were then furthered into a short list of attributes to be used in the visualizations.

**Table 1.** Overview and Assessment of Design Characteristics From the Literature Review (the So-Called Attributes) That Were Used in the Pilot and Were Evaluated in the First Survey.

*Attribute*	*Patient Room*	
	Client	Professional
*Materialization*	+	++
*Floor plan*	++	+
*Color scheme*	++	++
*Deep windowsills*	+	-
*Window:* Height	++	++
*Window:* Width	++	++
*Type doors:* Sliding*/hinging*	++	++
*Finishing ceiling*	+/−	+
*Type wall (pattern)*	+	++
*Type wall (translucent)*	++	+
*Type of floor covering*	+	+
*Temperature*	+	++
*Ventilation*	++	+
*Daylight (luminance)*	++	++
*Dimmers functional lights present*	++	++
*Dimmers mood lights*	+	+
*Possibility open/close windows*	++	++
*Rel*ative humidity	+/−	+
*Sunscreen position*	++	+
*Configuration of furniture*	++	++

### DCEs

**Figure 1.**
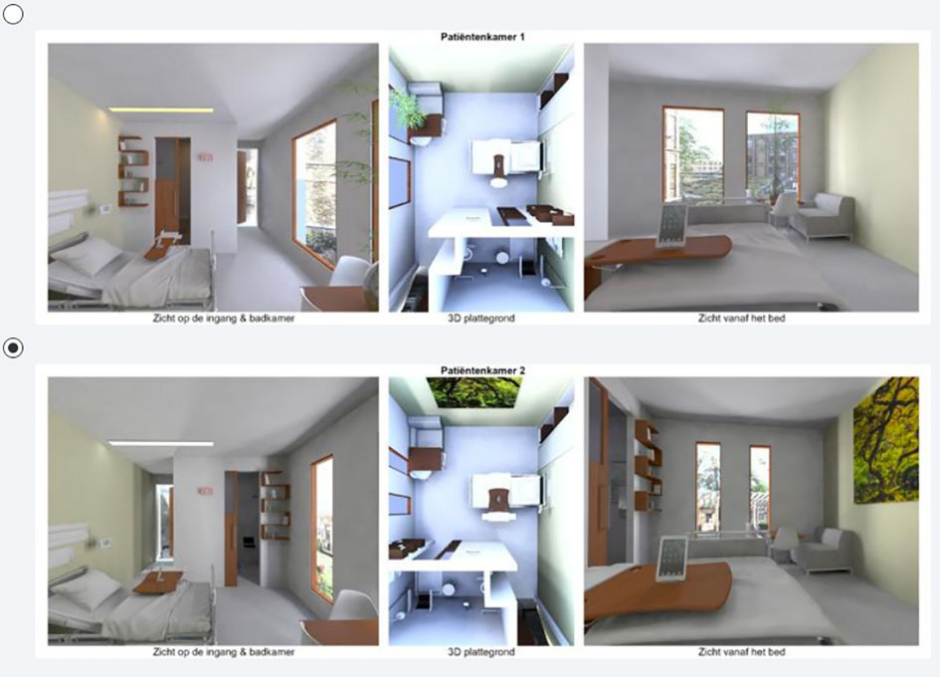
Example 1 of a discrete choice experiment for a (future) patient room. Top row shows a patient room with, for instance, an urban view, a small bedside table, an open wooden door, and the patient being sheltered against a direct view from the hallway (visual privacy). Bottom row shows a patient room with direct view to the hallway (safety), a large bedside table, and narrow instead of wide vertical windows with views to a pergola. The color of the lamps differs: The top row displays warm lighting and the bottom row displays cool lighting.

**Figure 2. fig2-1937586720937995:**
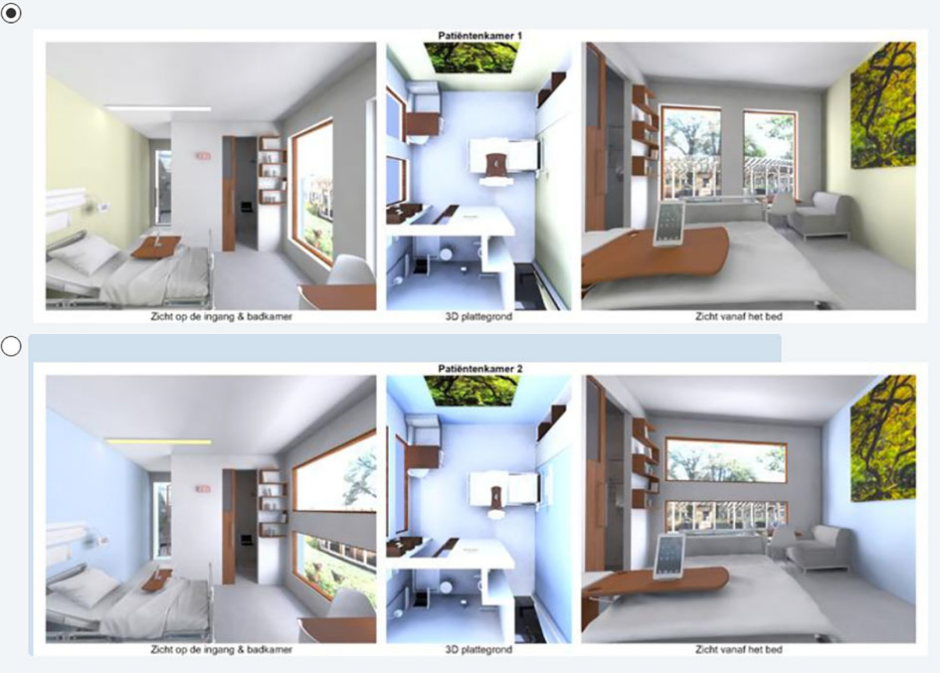
Example 2 of a discrete choice experiment for a (future) patient room. Both alternatives show a patient room with, for instance, a view to a pergola and direct sight lines (no visual privacy) to a transparent door. The door is closed in the top row and open in the bottom row. The bedside table also differs between the two hypothetical patient rooms. The room in the top row has wide vertically oriented windows, and the bottom row shows the more preferred option of a panoramic view.

Following the outcomes of the pilot, it was decided to use the attributes in Table 2 in the DCEs for the patient rooms. The attributes were then further conceptualized and visualized in Autodesk Maya 2016 (Version Autodesk Maya SP2) (2016) as displayed in Figures 1 [Fig fig2-1937586720937995]–3. To obtain sufficient statistical power, eight versions of seven DCE were made, following a receipt derived through optimization in SAS (see Kuhfeld, 2010). The SAS receipt was then edited in Maya to make different render layers in Maya. From each render layer (profile), two different renderings were created from different camera positions and combined with an overview of the floor plan in a visual display. In total 336 renderings were created as there were eight versions of seven questions with two alternatives consisting of three renderings each per room. These renderings were then combined in Adobe Premiere Pro.

**Figure 3.**
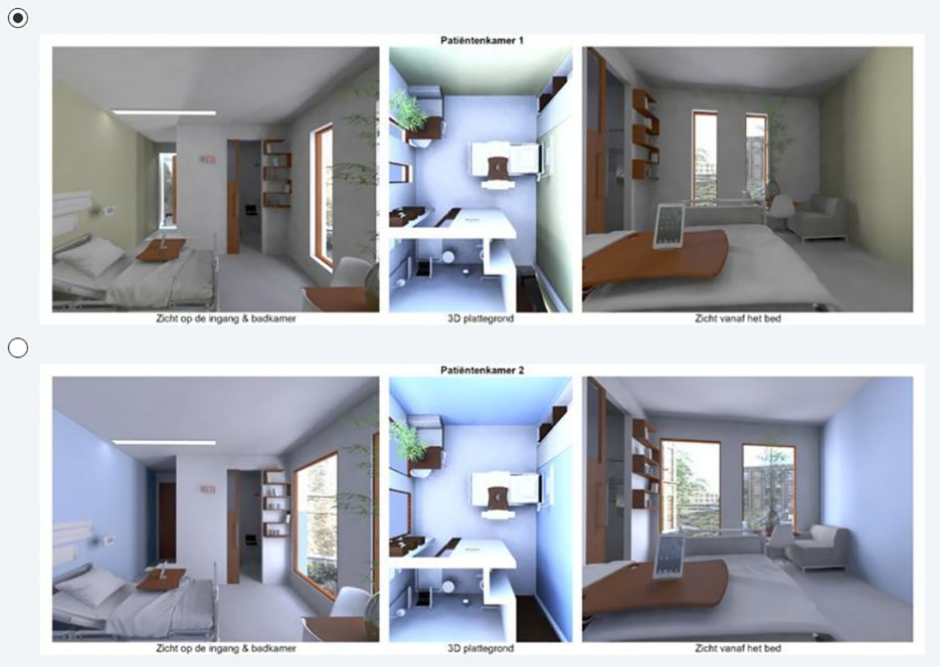
Example 3 of a discrete choice experiment for a (future) patient room. Both alternatives show a patient room with, for instance, an urban view and direct sight lines (no visual privacy) to a wooden door. The door is open in the top row and closed in the bottom row. The bedside table also differs between the two hypothetical patient rooms. The room in the top row has narrow, vertically oriented windows, and the bottom row has wide, vertically oriented windows. Rooms differ also in color (warm vs. cold). Both rooms have cold artificial lighting.

**Table 2.** Design Characteristics as Used in the Discrete Choice Experiments for the Patient Room.

*Attributes Patient Room*	*Level 1*	*Level 2*
*View toward bed*	Private (facing the wall)	Safe (facing hallway)
*View toward door open/close*	Door open	Door closed
*View door of glass/wood*	Wooden door	Glass door
*Door type*	Sliding door	Hinged door
*Orientation of windows*	Horizontal windows	Vertical windows
*Depth of window*	Deep	Shallow
*Color of wall*	Warm	Cool
*Color of lamps*	Warm, (4,000 K)	Cool (10,000 K)
*Quantity of daylight penetration*	Wide windows	Narrow windows
*Outside view*	Urban view	View to greenery (Pergola)
*Inside view to vegetation*	Vase and bamboo	Painting
*Size of bedside table*	Large	Small

### Procedure

Each respondent was asked to fill out a set of seven DCE questions and asked to indicate the most preferred patient room (or for medical professionals the one they thought best meets the patient’s needs). In the online survey program Qualtrics, respondents were therefore randomized into one of the eight versions of the DCE. Depending on the answer to whether or not respondents currently work or had work in the past 10 years as medical professional in a hospital, respondents were routed in a different way. For nonprofessionals, collected demographic information included age, education, and cultural background; for medical professionals, limited questions about demographic information were asked (e.g. age, gender) and more detailed information about working experience was collected.

The survey was voluntary, and at the start of the survey, it was explained that by continuing the survey, participants gave permission to use their answers for scientific purposes. The data set was anonymous and the Dutch Code of Conduct for Medical Research allows the use of anonymous data for research purposes, without an explicit informed consent (Stichting Federatie van Medisch Wetenschappelijke Vereningen, 2005).

### Data Analyses

Main effects were investigated in SAS Version 9.3 separately for patients (nonmedical professionals and therefore also referred to as laypersons) and respondents who currently are medical professionals or worked as medical professionals in the past 10 years. The conditional logit model was used to analyze the choice among the two vignettes (configurations) as a function of the attributes of the alternatives. In SAS, the PHREG procedure was used after preliminary data processing to fit a conditional logit model. The PHREG procedure fits the Cox proportional hazard model to survival data and the partial likelihood of Breslow has the same form as the likelihood in a conditional logit model as Kuhfeld (2010) explains. This model was used to analyze the influence of the attributes. The PHREG procedure uses the Hazard ratio (HR) as an effect measure and Firth corrected 95% confidence intervals (CI) were requested. A threshold of *p* < .05 was used in significance testing of the main effects. The Multinomial Discrete Choice (MDC) procedure was then used to estimate choice probabilities per attribute level and these were displayed to support interpretation. All preparations for data analyses in SAS were done in SPSS Version 24. SPSS was also used to obtain descriptive information.

### Reflexive Discussion With a Patient

The outcomes of the study were discussed with a hospitalized patient and enhanced the understanding of the outcomes. This patient was repeatedly hospitalized over an extended time and available through the social network of the researchers.

### Respondents

Social media and network were used to collect data including the publication of a own website, snowballing, Facebook, and LinkedIn. Since part of the social network may not master Dutch, the survey was also made available in English and French. During a stay in France, the survey was also actively promoted near a local hospital by distributing flyers.

## Results

Of the 212 respondents who started the questionnaire, 200 persons filled out the Dutch version of the survey, 7 filled out the French version, and 5 persons the English version. Of those who accessed the questionnaire, 204 continued upon the question that asked for their consent. Their average age was 37.9 years (95% CI [36.0, 39.9]). In total, 38.4% was male and 61.6% was female. Nearly 12% were currently active as a medical professional in an hospital, most were not (n = 178). However, among the 178 who were not working in a hospital, there were 56 who worked in the past 10 year as a medical professional. That means that 57.5% (n = 122) had never worked as a medical professional in the past 10 years and were considered patients. Of these persons, 42.1% (n = 51) was ever hospitalized for more than 1 day. For most of them (n = 38), this was more than a year ago. For about half of the nonmedical professionals, 51.7%, the most recent visit for to an outpatient consultation was more than 1 year ago.

### DCEs for the Patient Room

The analyses of the DCEs were stratified according to whether or not a respondent was a patient or a medical professional. Based on the choice patterns from the patient group (see Table 3), there were several design characteristics important in making a choice. Patients preferred a small side table over a bigger one. This can be inferred from the HR being significantly larger than 1.0. Indeed, the HR is 1.35 meaning that patients choose the smaller one, 35% more often chosen than they choose the bigger one. A HR significantly smaller than 1.0 would mean that the reference level is preferred. When the HR is not significantly different from 1.0, and thus the 95% CI holds the 1.0, then there is no preference for either of the two levels.

**Table 3.** Patients’ Valuation of Design Characteristics for Hospital Room From Patients’ Perspective.

Attribute	***B***	*SE*	**χ^2^**	***p***	HR	95% CI HR
Door type (Ref: *Sliding door*)						
Hinged door	0.074	.125	0.350	.55	1.077	[0.842, 1.378]
Size bedside table (Ref: *Large*)						
Small	0.299	.134	49.95	.03	1.348	[1.039, 1.755]
Outside view greenery (Ref: Urban)						
Pergola	0.206	.143	20.84	.15	1.229	[0.931, 1.630]
Inside view vegetation (Ref: Vase and bamboo)						
Painting	0.087	.134	0.423	.52	1.091	[0.840, 1.418]
Daylight penetration (Ref: Small windows)						
Wide windows	243.9	.187	1,710	<.01	11.459	[8.071, 16.858]
Depth of window (Ref: *Shallow*)						
Deep	0.230	.128	32.41	.07	1.259	[0.982, 1.619]
Orientation of windows (Ref: Vertical)						
Horizontal	0.682	.134	260.6	<.01	1.978	[1.529, 2.580]
View toward door (Ref: Closed)						
Open	0.298	.132	51.15	.02	1.347	[1.042, 1.746]
Color of wall (Ref: Cold)						
Warm	0.063	.137	0.209	.65	1.065	[0.814, 1.394]
View door (Ref: *Wooden*)						
Transparent	0.102	.133	0.592	.44	1.108	[0.855, 1.439]
Color of lamps (Ref: 4,000 K—warm)						
10,000 K—cold	0.118	.137	0.739	.39	1.125	[0.86, 1.475]
View toward bed (Ref: Private—facing the wall)						
Safe—facing hallway	0.062	.130	0.227	.63	1.064	[0.825, 1.373]

*Note*. Italics mark different reference levels than with medical professionals. HR = Hazard ratio; CI = confidence interval.

Both patients (Table 3) and medical professionals (Table 4) were particularly fond of the wide, horizontal windows. This is an important finding, as it shows that architectural design characteristics are important. These findings cannot be explained by a difference in the amount of daylight access. Indeed, the preference for the wider window does show that daylight access is important. However, the vertical windows would provide as much daylight access as the horizontal.

**Table 4.** Medical Professionals’ Valuation of Design Characteristics for Hospital Room From Patients’ Perspective Including Interaction Effects.

**Attribute**	***B***	*SE*	χ^2^	***p***	**HR**	**95% CI HR**
Door type (Ref: *Hinged door*)						
Sliding door	0.143	.147	0.939	.33	1.153	[0.866, 1.539]
Size bedside table (Ref: *Small*)						
Large	0.130	.159	0.668	.42	1.138	[0.836, 1.555]
Outside view greenery (Ref: Urban)						
Pergola	0.683	.177	149.9	<.01	1.981	[1.412, 2.823]
Inside view vegetation (Ref: Vase and bamboo)						
Painting	0.133	.162	0.663	.42	1.142	[0.832, 1.571]
Daylight penetration (Ref: Small windows)						
Wide windows	205.7	.211	948.1	<.01	7.821	[5.266, 12.092]
Depth of window (Ref: *Deep*)						
Shallow	0.295	.161	33.62	.07	1.343	[0.984, 1.844]
Orientation of windows (Ref: Vertical)						
Horizontal	0.673	.172	152.2	<.01	1.960	[1.407, 2.761]
View toward door (Ref: Closed)						
Open	−0.163	.196	0.692	.41	0.849	[0.579, 1.243]
Color of wall (Ref: Cold)						
Warm	0.308	.160	37.20	.05	1.360	[1.000, 1.865]
View door (Ref: *Transparent*)						
Wooden	−0.956	.280	117.0	<.01	0.384	[0.218, 0.657]
Color of lamps (Ref: 4,000 K—warm)						
10,000 K—cold	0.292	.165	31.41	.08	1.339	[0.972, 1.853]
View toward bed (Ref: Private—facing the wall)						
Safe—facing hallway	0.088	.157	0.310	.578	1.091	[0.804, 1.484]
Interaction View Door × View Toward Door (Ref: Transparent, closed door)						
Wooden, open door	115.0	.346	110.7	<.01	3.158	[1.619, 6.281]

*Note*. Italics mark different reference levels than with patients. HR = Hazard ratio; CI = confidence interval.

Finally, patients preferred the door to the hallway to be open instead of closed. This characteristic was included as it was argued that it would add to a sense of safety in the eyes of a patient. This might be particularly so in the situation of single patient rooms, as was here the case.

It is interesting to note the differences between the valuation by the patients themselves and what the patients are believed to prefer according to medical professionals. Aside from the amount of daylight access and the horizontal windows, the medical professionals underscored the importance of an outside view to greenery, a warm color scheme instead of a cold one (HR = 1.45 without interactions), and a more bluish artificial lighting color (10,000 K instead of the more yellow colored light of 4,000 K). These outcomes are very much consistent with the literature (see, for instance, Herweijer-van Gelder, 2016).

### Interactions

At forehand, there was one interaction anticipated, that is, the interaction between whether or not the door of the patient room was transparent and whether or not the door was open or closed. The latter was added as an attribute as it may interfere with a sense of safety that might be particularly important with the more recent introduction of single patient rooms only. Single patient rooms are particularly important to avoid cross contamination but, as it was thought, may introduce feelings of discomfort to the patient, because the presence of other patient may be also helpful at times. Another feature would be that a patient could hear medical professionals passing by, which might also increase a sense of liveliness.

Indeed, this interaction was found to be significant, but only for the medical professionals. Patients preferred an open door anyway (HR = 1.2, p < .05). Medical professionals however thought that a patient would only prefer an open door in case of a nontransparent wooden door (HR = 3.2, 95% CI [1.62, 6.28], p < .001). It seems that they were more concerned with the patient’s privacy. Other interactions were not found to be significant at p < .01, which was used as a threshold for significance testing for interaction effects.

### Most Preferred Room

**Figure 4.**
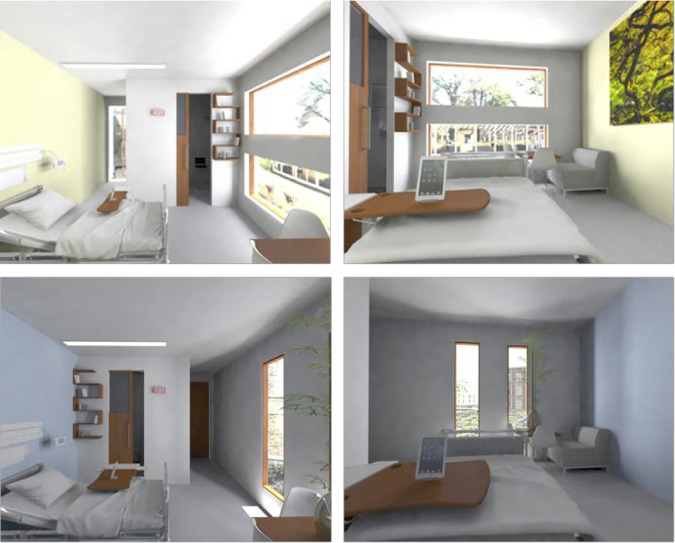
Top row shows the most preferred room according to patients, and the bottom row shows the least.

To visualize the outcomes of the present study from the perspective of the patient, Figure 4 shows the most and least preferred single patient room.

## Summary and Discussion

To some extent, patients are currently involved in the design of hospitals but given the results of studies by Elf et al. (2015) and their plea for shared-decision-making and collaborative design processes with representatives from healthcare, construction sector, and architecture based on evidence and end users’ perspectives, it is clear that such is not yet common practice. Increasing specialization and technological developments add to the increasing complexity of design processes. Using 3D design models, this study aims to better involve patients by investigating what physical environmental characteristics in hospital patient rooms are valued by patients.

### Panoramic View

Patients and medical professionals consistently choose for both hospital rooms with the highest amount of daylight access. The importance of daylight access, an attribute that also appeared in the text analyses of the abstracts identified in the literature search, is generally agreed upon in literature (Huisman et al., 2012). What this study adds is that the orientation of the windows matters as well. Horizontal windows, allowing for a panoramic view, were twice as much chosen than were vertical windows. Previously, in housing, it was found that the orientation of the windows matters, but unlike the current study, in residential housing people prefer vertically oriented windows (Riccardo et al., 2012). Given the consistent findings, it may well be that patients might prefer horizontally oriented windows, because being a patient and whether one is bed-bounded or not, the panoramic view is more attractive, because it is easier to follow some events across the window as a patient in a reflexive interview told. A panoramic view may support directed attention, and in the presence of high amounts of daylight, this might be the kind of environment that might perceived as coherently ordered and of substantial scope, supporting an effortless mode of functioning (von Lindern et al., 2017). In terms of the attention restoration theory (ART; Hadavi et al., 2015; Kaplan, 1995; von Lindern et al., 2017), an environment is restorative if rich in fascinating features.

***What this study adds is that the orientation of the windows matters as well. Horizontal windows, allowing for a panoramic view, were twice as much chosen than were vertical windows***.

### Safety and Privacy Versus Connecting

Another important finding concerns patients’ preferences for an open door. In the design of the DCEs, safety and privacy were considered important, echoing the recent developments in hospital design to prefer single patient rooms to multiple patient rooms (Van de Glind et al., 2007). For the patient room, the door was either open or closed (view to door) and could be either transparent because of the use of glass or not transparent and materialized with a wooden print (view door), and the view toward the bed could be either private because the bath room blocked off the sight line from the door to a patient’s bed or the bed would be visible directly from the entrance. The latter situation was chosen as it may well be that such would give a patient a sense of safety, if staff would pass by. Patients expressed a significant preference for an open door, whether the door was transparent or not. Furthermore, patients did not mind that they were visible from the entrance as well. Since no interactions here were significant, the interpretation is that patients would choose for a situation where they would be able “to hear” or “stay connected with” the hallways. This finding diverges from what Patterson et al. (2017) reported using a simulated room combined with printed room layouts to highlight differences. Their results lend support to the interpretation that visibility of the hallway, in the current study the open door, was important to provide them with a sense of safety and security, However, whereas they found mixed perceptions as to whether or not people preferred the use of a privacy curtain, we found that people preferred an open door irrespective of the level of privacy corroborating their report of participants’ need to stay connected to people and the outside world. As with the panoramic view, this may well fit the ART theory that too strong isolation does not add to a restorative environment.

***Patients expressed a significant preference for an open door, whether the door was transparent or not***.

***the interpretation is that patients would choose for a situation where they would be able “to hear” or “stay connected with” the hallways***.

### Evidence-Based Design

In comparing differences between what patients valued and what medical professionals thought patients would value, it is striking that medical professionals thought that patients would prefer a patient room with outside view to a pergola, which compares to an outside view to greenery, and would prefer warm colored walls over cold colored walls and cold colored light from lamps over warm colored artificial lighting. Patients did not make these kinds of choices though. The kind of choices the medical professionals thought patients would prefer is highly consistent with what has been reported in the scientific literature and is often referred to as evidence-based design (Huisman et al., 2012). These attributes were also identified through the text analyses of the literature search. The discrepancy between what patients chose themselves and what medical professionals think patients prefer is an important finding of this study. Indeed, one might argue that it reflects the aforementioned problems that in the existing literature, environmental design factors are poorly conceptualized, that the same concepts are operationalized in different ways between medical and technological sciences (Aarts et al., 2016; van Hoof et al., 2010), and that such research typically lacks sufficient details for replication and comparisons across studies. Moreover, a major problem is that many of these studies do not use a quantitative approach in which multiple attributes are concurrently assessed in a visual display—as in the present study with DCEs (van Oel & van den Berkhof, 2013). Indeed, studies that use a more holistic approach typically use a qualitative research approach, and outcomes of this kind of studies fell short in providing strong evidence (Huisman et al., 2012).

This study is therefore important as it shows, empirically, that patients may make different choices if the rooms are better conceptualized and thus visualized and if multiple design features are assessed as configuration rather than using a sequential, “one-design-characteristic-after-another” approach.

***This study is therefore important as it shows, empirically, that patients may make different choices if the rooms are better conceptualized and thus visualized and if multiple design features are assessed as configuration rather than using a sequential, “one-design-characteristic-after-another” approach***.

### Limitations and Recommendations

There are several limitations to mention that might be addressed in further research. Important to mention is that the current study used snowball sampling of respondents. It would have been better if the survey was distributed in a more systematic way, for instance, in collaboration with hospitals. Due to time constraints, this was not possible, and respondents were therefore sampled as described.

A second limitation might be that the use of an online survey may not sufficiently approach the real situation. Virtual reality (VR) might be a better alternative, although the disadvantage is that this may be too overwhelming at first sight. Maybe a combination of the current approach and further validation of the significant attributes through VR is most convenient. First, using an online survey can highlight the most important features, and then VR can be useful to investigate whether the choices really reflect one’s preferences. For instance, the preference for a horizontal orientation of the windows may have been different, had we used immersive visualization. Indeed, the use of immersion would be particularly interesting because it would allow us to more closely mimic the sight lines from the head of the bed as well as from a laying, sitting, and standing position. In the current study, the choice to have one of the camera positions placed at the head of the bed may have strengthen the preference for a panoramic view at the dispense of the bedside table. This camera position introduced a distortion of the visualization of the bedside table, as it was close to the camera. Immersion would certainly have overcome this issue.

Finally, as a recommendation, for a better perception of the rooms, it is important to consider the use of immersive visualization. Here, we used 3D visualization but as it was offered in an online survey, the perception would be of a 2D display. This may have affected the current outcomes. Instead, we discussed the outcomes with a patient with extensive experience. This provides much insight about why the panoramic view is so important. These kinds of follow-up interviews with patients and others may be very important to better understand the reported preferences. For instance, one may wish to learn whether or not patients like to have the door open to stay in touch with others reflects a need for safety or not.

## Implications for Practice

- Communication with users about future design options is more easy using 3D renderings than relying on verbalizations.
- Combining 3D visualizations and DCEs in a survey can be a way to involve (future) patients and healthcare practitioners, respectively, in the design process and learn about their preferences.
- In designing from a patient’s perspective, one need to involve (future) patients, as for medical professionals, it is very difficult to stand in their shoes and avoid interferences with one’s own preferences.
- In a single patient room, a panoramic outside view may help patients to feel connected to the outside world.
- Patients also preferred an open door, irrespective of its transparency, also suggesting that it helps them to stay connected.
